# Bosutinib high density lipoprotein nanoformulation has potent tumour radiosensitisation effects

**DOI:** 10.1186/s12951-023-01848-9

**Published:** 2023-03-21

**Authors:** Pouya Dehghankelishadi, Parisa Badiee, Michelle F. Maritz, Nicole Dmochowska, Benjamin Thierry

**Affiliations:** 1 Future Industries Institute and ARC Centre of Excellence Convergent Bio-Nano Science and Technology, University of South Australia, Mawson Lakes Campus, Adelaide, SA 5095 Australia; 2 UniSA Clinical and Health Sciences, University of South Australia, City West Campus, Adelaide, SA 5000 Australia

**Keywords:** Cell cycle, Radiosensitiser, High density lipoprotein nanoparticle, Bosutinib, Reformulation

## Abstract

Disruption of the cell cycle is among the most effective approach to increase tumour cells’ radio-sensitivity. However, the presence of dose-limiting side effects hampers the clinical use of tyrosine kinase inhibitors targeting the cell cycle. Towards addressing this challenge, we identified a bosutinib nanoformulation within high density lipoprotein nanoparticles (HDL NPs) as a promising radiosensitiser. Bosutinib is a kinase inhibitor clinically approved for the treatment of chronic myeloid leukemia that possesses radiosensitising properties through cell cycle checkpoint inhibition. We found that a remarkably high bosutinib loading (> 10%) within HDL NPs could be reliably achieved under optimal preparation conditions. The radiosensitisation activity of the bosutinib-HDL nanoformulation was first assessed in vitro in UM-SCC-1 head and neck squamous cell carcinoma (HNSCC) cells, which confirmed efficient disruption of the radiation induced G_2_/M cell cycle arrest. Interestingly, the bosutinib nanoformulation out-performed free bosutinib, likely because of the specific affinity of HDL NPs with tumour cells. The combination of bosutinib-HDL NPs and radiotherapy significantly controlled tumour growth in an immunocompetent murine HNSCC model. The bosutinib-HDL nanoformulation also enhanced the radiation induced immune response through the polarisation of tumour associated macrophages towards proinflammatory phenotypes.

**Supplementary Information:**

The online version contains supplementary material available at 10.1186/s12951-023-01848-9.

## Introduction

In contrast to standard chemoradiation treatments based on cytotoxic drugs, radiosensitisers should ideally enhance radiobiological effects selectively in tumour tissues, and consequently both improve oncological outcomes and minimise side effects in healthy organs at risk. Disruption of the cell cycle is among the most effective approach to increase tumour cells’ radio-sensitivity because tumour cells defective in the G_1_ cell cycle checkpoint rely mainly on the G_2_/M cell cycle checkpoint to avoid premature mitosis. Disruption of the G_2_/M cell cycle checkpoint forces irradiated cells into mitosis with a significant burden of DNA damages, which can lead to mitotic catastrophe and cell death [1]. WEE1 kinase is a key regulator of the G_2_/M cell cycle checkpoint, and small molecule WEE1 inhibitors such as AZD1775 have been shown to be potent radiosensitisers in many cancer types [2, 3]. Another potent WEE1 kinase inhibitor is bosutinib (Additional file 1: Fig S1) [4, 5], which has been previously shown to potentiate the effects of DNA damaging agents and radiation [6]. Bosutinib is a BCR-ABL1 tyrosine kinase inhibitor approved for the treatment of chronic myeloid leukemia but, despite its somewhat favourable toxicity profile compared to other potent tyrosine kinase inhibitors, its use remains commonly associated with dose-limiting side effects including gastrointestinal toxicity and myelosuppression [7].

The unfavourable toxicity profile of bosutinib, and more generally of small molecule tyrosine kinase inhibitors targeting the cell cycle, is mainly associated with systemic distribution to off-target organs and hampers their application as radiosensitisers. The benefit of formulating an aurora B kinase inhibitor within PLGA-PEG nanoparticles was recently demonstrated, yielding potent tumour responses in several preclinical models at tolerable doses [8]. The promising toxicity data observed preclinically was subsequently confirmed in a Phase I trial, confirming the potential of tyrosine kinase inhibitor nanoformulation [9]. Among candidate nanocarriers targeting solid tumours, high density lipoprotein nanoparticles (HDL NPs) present distinctive advantages [10]. Reconstituted HDL NPs are synthetic versions of natural HDLs, a lipoprotein involved in cholesterol transport that has been actively investigated for the delivery of a range of therapeutics including small molecule drugs and RNA therapeutics [11, 12]. Importantly, HDL NPs are naturally targeted to the tumour tissue through interactions with scavenger receptors such as SR-B1 that are overexpressed in both cancer cells and tumour associated macrophages (TAMs) [13–17]. Biodistribution studies in animal models yielded 3.6 and 2 times higher accumulation of HDL NPs in tumour tissues compared to liposomes and PEGylated liposomes, respectively [18]. In addition, the recent TARGET phase II clinical trial confirmed the effective accumulation of radio-labelled HDL NPs in oesophageal tumours [19, 20]. The natural tumour homing capabilities of HDL NPs combined to their excellent safety profile suggests their potential for addressing the dose-limiting toxicity of tyrosine kinase inhibitors targeting the cell cycle.

A bottleneck in the use of HDL NPs as delivery vehicles for small molecule drugs to solid tumours is that in most cases, only low-to-moderate amounts of therapeutic payloads can be incorporated with the lipid core [18, 21, 22]. The loading depends on both the formulation parameters and the physicochemical properties of the drug and its affinity with the HDL NPs. Screening for WEE1 kinase inhibitors compatible with HDL nanoformulation, we determined that bosutinib has a high affinity with HDL NPs, enabling ultra-high loading greater than 10% (w/w). In contrast, the potent AZD-1775 kinase inhibitor yielded only neglectable loading in the HDL NPs (< 0.1%).

Building on this finding, we endeavoured to comprehensively determine the radiosensitisation properties of the bosutinib-HDL nanoformulations. The effects of combined bosutinib-HDL nanoformulation and radiation treatment on cell cycle pattern, viability, proliferative index and apoptosis were evaluated in vitro in the UM-SCC-1 head and neck squamous cell carcinoma (HNSCC) cell line. The in vivo efficacy of the bosutinib-HDL nanoformulation was validated in an immune-competent heterotopic MOC-1 HNSCC tumour bearing murine model. These studies established the potent radiosensitising properties of the bosutinib-HDL nanoformulation, warranting further preclinical investigations to fully establish both its therapeutic efficacy and safety profile.

## Materials and methods

### Materials

1,2-dipalmitoyl-sn-glycero-3-phosphocholine (DPPC) was purchased from Avanti Polar Lipids (USA). ApoA-1 mimicking peptide (22A) with sequence of PVLDLFRELLNELLEALKQKLK was synthesized by Genscript (USA) [23]. Bosutinib was obtained from MedChem Express (USA). ^18^F-fluorodeoxyglucose (^18^F-FDG) was generously provided by the Molecular Imaging and Therapy Research Unit (MITRU), South Australian Health and Medical Research Institute (SAHMRI).

### Preparation of bosutinib-HDL NPs

The bosutinib-HDL NPs were prepared using a film hydration technique. DPPC and bosutinib (5 DPPC: 2 bosutinib w/w) were dissolved in methanol in a round bottom flask. The methanol was evaporated using a rotary evaporator. The resulting thin film was hydrated with a ApoA-1 mimicking peptide solution (2.5 peptide: 5 DPPC w/w) in phosphate buffered saline (PBS). The solution was sonicated for 15–30 min followed by 4 rounds of thermal cycles at 25 °C (5 min) and 50 °C (5 min). The resulting nanoparticle solutions were concentrated and washed twice using Amicon centrifuge inserts (molecular weight cut-off (MWCO), 10 kDa). The final nanoparticle solutions were filtered through a 0.22 µm polyethersulfone (PES) filter. The bosutinib-HDL NPs were lyophilized (ModulyoD Freeze Dryer, Thermo-Fisher, USA) and stored at − 20 °C. Reconstituted bosutinib-HDL NP samples were filtered with 0.22 µm filter before use.

### Physicochemical characterisation of bosutinib-HDL NPs

Dynamic light scattering (Zetasizer 3000 HS, Malvern Instruments Ltd) was used for analysing the hydrodynamic size of the bosutinib-HDL NPs. The NPs morphology was analysed by transmission electron microscopy (JEM-2100F-HR, Jeol, Japan). Negative staining was performed with phosphotungstic acid (1%). The peptide content of the bosutinib-HDL NPs was quantified using the Pierce™ BCA Protein Assay Kit (Pierce Biotechnology, USA) according to the manufacturer’s protocol. The bosutinib content was analysed using HPLC (Agilent 1200 Liquid Chromatograph—1200 FLD/DAD, C18 Eclipse Plus column) after the dissolution of the bosutinib-HDL NPs in 75% methanol solution. The mobile phase gradient consisted of 10% acetonitrile (0.1% trifluoroacetic acid) and 90% Milli-Q water (0.1% trifluoroacetic acid), which changed over 8 min to 90% acetonitrile (0.1% trifluoroacetic acid) and 10% Milli-Q water (0.1% trifluoroacetic acid) at 2 mL/min flow rate (detection wavelength 265 nm).

### Cell culture

UM-SCC-1 cells (EMD Millipore, USA) were cultured in 75 cm^2^ tissue culture flasks in RPMI Media (Gibco, UK) supplemented with 10% FBS (Gibco, UK), 1% L-glutamine (Gibco, UK) and 1% streptomycin/penicillin (Gibco, UK) at 37 °C, in a humidifying incubator (with 5% CO_2_). The doubling time of UM-SCC-1 cells is 22 h [24]. UM-SCC-1 cells stably expressing the fluorescent ubiquitination based cell cycle indicator (fucci) system, were used for cell cycle analysis [25]. The fucci constructs were provided by RIKEN BRC through the National Bio-Resources Project of the MEXT/AMED, Japan. U87 glioblastoma cells (ATCC) were maintained in MEM media supplemented with 10% FBS (Gibco, UK) and 1% streptomycin/penicillin (Gibco, UK). Mouse Oral Squamous Cell Carcinoma cell line (MOC-1, Kerafast cell bank) were maintained in IMDM media (Gibco, UK) supplemented with 33.1% Hams Nutrient Mixture (Gibco, UK), 5% FBS (Gibco, UK), 1% streptomycin/penicillin (Gibco, UK), insulin (5 µg/mL, Sigma), hydrocortisone (40 ng/mL, Sigma) and epidermal growth factor (EGF, 5 ng/mL, EMD Millipore). All cells were maintained, and experimentation was performed at 37 °C. All cell lines were passaged when at 80% confluency.

### Bosutinib uptake analysis

To assess bosutinib uptake, 4 × 10^6^ UM-SCC-1 cells were seeded in 75 cm^2^ tissue culture flasks. 24 h after cell seeding, the cells were treated with either bosutinib-HDL NPs or free bosutinib (equivalent to 10 µM based on bosutinib concentration) in full media. After 5 h incubation, the cells were washed 3 times with PBS, trypsinized and resuspended in 500 µL acetonitrile. The samples were sonicated 50 s with a probe sonicator. To extract bosutinib, 4 mL ethyl acetate:hexane (90:10, v/v) was added to each sample and incubated for 15 min. The samples were centrifuged at 1500 g for 5 min and 4 mL of supernatant was transferred to another tube and evaporated to dryness using a Christ RVC 2–25 CDplus Speed Vac. The dried samples were finally resuspended in 500 µL methanol and bosutinib was quantified using HPLC.

### Investigation of radiosensitisation properties of bosutinib-HDL NPs in UM-SCC-1 cell line

Cell cycle and caspase 3/7 activity as a marker for early apoptosis were analyzed 24 h after irradiation as early markers of radiobiological responses while ATP content and proliferative index (ki67 staining) were measured at a later time point (48 h) to reflect the expected mechanisms.

The viability of UM-SCC-1 cells was investigated 48 h after radiation treatment using the CellTiter-Glo^®^ assay according to the manufacturer’s protocol. Briefly, 7.5 × 10^3^ UM-SCC-1 cells per well were seeded in 96 well plate. The following day, cells were treated with HDL NPs, bosutinib-HDL NPs or free bosutinib (5 µM based on bosutinib concentration (1.7 µg/cm^2^)) in full media. After 2 h, cells were irradiated (6 Gy) with an RS2000 irradiator (Rad Source Technologies Inc, USA). The radiation dose was selected based on previous radiosensitisation studies focused on cell cycle check point inhibitors [3, 26]. For the radiosensitisation experiment with U87 cells, similar experimental conditions were used except that U87 cells were seeded at 4000 cells per well and the ATP content was measured 72 h after irradiation.

4 × 10^4^ UM-SCC-1 or fucci-UM-SCC-1 cells per well were seeded in ibiTreat µ-Slide 8 Well (Ibidi GmbH, Germany) for apoptosis and cell cycle analyses, respectively. The bosutinib treatment concentration was 5 µM (0.5 µg/cm^2^). The radiation protocol described above was used. For apoptosis analyses, the caspase 3/7 activity was measured using CellEvent™ Caspase-3/7 Green Detection Reagent (Thermo-Fisher, 1:1000 v/v in full media). For the cell cycle experiment, no cell cycle synchronisation was induced. Cells were imaged (24 h after radiation) using 10 × objective in the FITC and/or Texas Red channels using a Confocal Laser Scanning Microscope (Zeiss, Germany). The numbers of cells per image were automatically counted with the NIS-Elements software (Nikon, Japan).

For Ki-67 immuno-staining, UM-SCC-1 cells were seeded in 6 well plates (2 × 10^5^ cells per well). The bosutinib treatment concentration was 5 µM (0.5 µg/cm^2^). The radiation protocol described above was used. Cells were trypsinized and fixed/permeabilized using eBioscience™ Intracellular Fixation and Permeabilization Buffer Set (USA) according to the manufacturer’s protocol (48 h after irradiation) and stained with Alexa Fluor® 647 anti-human Ki-67 antibody (Biolegend, USA). The cells were washed twice with PBS and an imaging flow cytometer Image Stream X (AMNIS, USA) was used for analyses. Flow cytometry data was processed using IDEAS^®^ 6.2 (EMD Millipore, USA) and FlowJo V10 (FlowJo, USA).

### Investigation of the radiosensitisation properties in MOC-1 tumour syngeneic murine model

All in vivo experiments were performed according to the protocols approved by the SAHMRI animal ethics committee (application SAM-21–001). C57BL/6 mice 6 weeks old were obtained from the SAHMRI Bioresources breeding colony and were group-housed in specific pathogen-free conditions, according to SAHMRI’s animal care protocols under the standard diet.

Mice were injected subcutaneously with 1 × 10^6^ MOC-1 cells (50% v/v, Cultrex Basement Membrane Extract, Type 3) in the right flank. The flank region was inspected by palpation to confirm the presence of a tumour. When the tumour lump was detected, the length and width of the tumour were measured with a calliper to calculate the tumour volume, calculated using the formula V = A × b^2^ × 0.5 where A is the length and b is the measured width of the tumour. When tumour sizes reached 50–100 mm^3^, mice were assigned to the different treatment groups. Bosutinib-HDL NPs (20 mg/kg based on bosutinib concentration) or an equivalent dose of blank HDL NPs were administered by lateral tail vein injection, according to the treatment protocol depicted in Fig. 3-A. The irradiation was performed using an RS-2000 Irradiator, an X-ray irradiator working at 160 kV and 25 mA output. The animals were placed on a target map adjusted at shelf one. The dose rate for shelf one is ~ 1.09 Gy/min. Mice were irradiated for 2 min and 45 s (3 Gy, 6 times during treatment period, total radiation dose 18 Gy). Irradiation was performed 2 h after nanoparticle/drug treatment. The mouse body, except for the tumour site, was covered by a lead radiation shield. Tumour and body weight measurements were performed three times a week during the experiment. After completion of the treatment, tumour metabolic activities were assessed using ^18^F-FDG positron emission tomography (PET) imaging. Mice were fasted overnight and administered with ^18^F-FDG via intra-peritoneal injection [27]. PET imaging was performed with sub-millimetric resolution (0.7 mm), using the Albira PET-SPECT small animal scanner (Bruker Biospin, GmbH). The PET images were analysed using manually drawn regions of interest in the PMOD Imaging Suite (PMOD Technologies, USA) to calculate total lesion glycolysis (TLG%, Eq. 1) and standard uptake value (SUV%, Eq. 2) for each tumour. Mice were euthanized by CO_2_ inhalation, when there was a > 10% reduction in body weight relative to the starting body weight or if actively bleeding tumour ulcers were observed. At the experimental endpoint, tumours were harvested and dissociated for immune cell phenotyping.

1 $${\text{TLG }}\left( {\text{\% }} \right) = \frac{{{\text{Tumour activity }}\left( {\frac{{{\text{kBq}}}}{{{\text{cc}}}}} \right) \times {\text{tumour volume }}\left( {{\text{cc}}} \right)}}{{{\text{Activity injceted }}\left( {{\text{kBq}}} \right) \times {\text{Mice weight }}\left( {\text{g}} \right)^{ - 1} { }}} \times 100$$

2 $${\text{SUV }}\left( {\text{\% }} \right) = \frac{{{\text{Tumour activity }}\left( {\frac{{{\text{kBq}}}}{{{\text{cc}}}}} \right)}}{{{\text{Activity injceted }}\left( {{\text{kBq}}} \right) \times {\text{Mice weight }}\left( {\text{g}} \right)^{ - 1} { }}} \times 100$$

A toxicity study was performed in healthy (non-tumour-bearing) mice. C57BL/6 mice 6 weeks old were intravenously injected with either PBS or bosutinib-HDL NPs (20 mg/kg, IV). After two weeks, the mice were humanely killed by cardiac puncture. The blood samples were used for the analysis of key biochemical markers including alanine aminotransferase (ALT), aspartate aminotransferase (AST), creatine kinase (CK), alkaline phosphatase (ALP) and total bilirubin (3 mice per treatment group). Key organs including the liver, kidneys, lungs and spleen were collected and stained with hematoxylin and eosin.

### Flow cytometric analyses of immune cells in the tumour tissue

Harvested tumour tissues were mechanically minced and incubated with 300 U/mL collagenase, 100 U/mL hyaluronidase (Stem-Cell Technologies) and DNase I (0.1 mg/mL, Sigma) for 45 min at 37 °C and 400 rpm. The cell suspension was filtered through 70 µm cell strainers. The cells were washed and incubated with the viability stain 575 V (15 min, BD Biosciences), Fc blocked with CD16/CD32 antibody (5 min, BD Biosciences), and stained with a cocktail of antibodies (listed in Additional file 1: Table S1) in Brillant Stain Buffer (BSB, BD Biosciences). Flow cytometric data was acquired using a Fortessa flow cytometer (BD Biosciences) and analyzed with FlowJo V10 (FlowJo, USA).

### Statistical analysis

The data was analysed by analysis of variance (ANOVA) and Tukey post hoc test using GraphPad Prism 7 (GraphPad Software, Inc., USA), P values < 0.05 were considered significant. The data is represented as average ± standard deviation unless stated otherwise.

## Results and discussions

Discoid HDL nanoformulations offer several advantages for solid tumour drug delivery, such as intrinsic tumour homing properties and high tissue penetration. However, a drawback of discoid HDL NPs as a delivery platform is the limited drug loading capacity. The choice of a specific therapeutic payload, as well as the lipid composition of the HDL NPs are the key parameters that can be tuned to increase the drug loading capacity. DPPC was selected as the phospholipid component of the HDL NPs. DPPC has longer and hence more hydrophobic fatty acid tails compared to 1,2-dimyristoyl-sn-glycero-3-phosphocholine (DMPC) commonly used for the preparation of synthetic HDL NPs. The transition temperature of DPPC is 41 °C, which is optimal for the preparation of HDL NPs. The use of phospholipids with low transition temperatures in HDL nanoformulations leads to aggregation [22]. In addition, a low lipid transition temperature was also shown to be associated with lower circulation half time in pharmacokinetics studies in mice models [28]. In this work, the 22A ApoA-1 mimicking peptide was used as peptide component. Its in vitro/in vivo functionality has been demonstrated previously [21–23, 29, 30].

After trials of several potential WEE kinase/cell cycle checkpoint inhibitors, bosutinib was selected as the radiosensitising payload for loading into HDL NPs. For the preparation of bosutinib-HDL NPs, the bosutinib and DPPC were dissolved in methanol. Methanol was then evaporated to dryness to prepare a thin film of bosutinib and DPPC. Upon hydration of the film, hydrophobic bosutinib molecules and lipids nano-precipitates, forming the core of the HDL NPs. The bosutinib-lipid core is swiftly stabilized by ApoA-1 mimicking peptides dissolved in the aqueous phase. The bosutinib-HDL nanoformulation yielded a very high bosutinib loading of 11.4 ± 1.7% (w/w) (Table 1). In contrast, the potent WEE1 kinase inhibitor AZD-1775 yielded only very small loading (< 0.1% w/w loading) within HDL NPs in all tested experimental conditions. While several approaches have been proposed to increase drug loading within HDL NPs, for example through derivatisation or complexation with hydrophobic moieties [18, 31–33], the increased design complexity is a limitation towards translation. Contrastingly, the excellent loading obtained for bosutinib highlights the potential and benefits of the careful selection of the payload at the formulation design stage.

**Table 1:** Composition of bosutinib-HDL NPs and HDL NPs

	Bosutinib (%)	Peptide (%)	Lipid (%)
Bosutinib-HDL NPs	11.4 ± 1.7	76.2 ± 8.8	12.3 ± 9.6
HDL NPs	–	33.5 ± 8.3	66.2 ± 8.8

Data is reported as w/w %

**Fig. 1:**
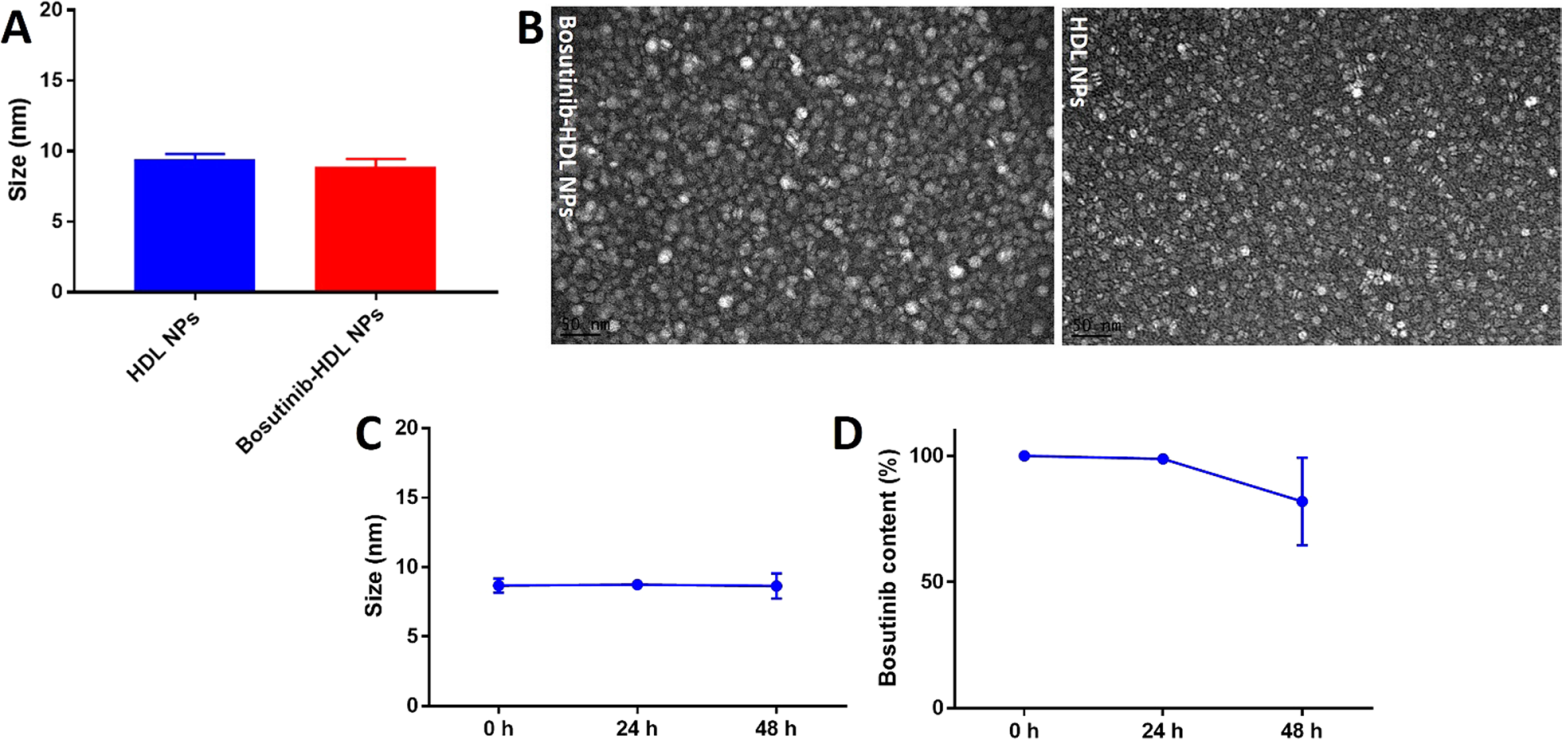
Hydrodynamic diameter, morphology and stability of bosutinib-HDL NPs. **A** Hydrodynamic diameter distribution and **B** TEM imaging of the bosutinib-HDL NPs and HDL NPs. Samples were negatively stained with 1% phosphotungstic acid. Scale bar is 50 nm. **C**, **D** Stability of bosutinib-HDL NPs (size and bosutinib content) in PBS at 37 °C (n = 3)

The hydrodynamic diameter of the bosutinib-HDL NPs prepared by this method was 8.9 ± 0.6 nm according to DLS measurement (Fig. 1A). The encapsulation of bosutinib did not change the hydrodynamic diameter compared to the HDL NPs (9.4 ± 0.4 nm). The discoidal morphology of the bosutinib-HDL NPs was confirmed using TEM imaging (Fig. 1B). TEM measurements confirmed DLS measurements, yielding an average size of 10 nm for the nanoformulation. The composition of the bosutinib-HDL NPs was notably different from that of the drug-free HDL NPs in terms of lipid and peptide contents. This likely originates in the substitution of some of the core lipid components by hydrophobic bosutinib molecules, leading to a reduction in the lipid content of the final formulation. The change in the core components of the particles has also translated to a change in the peptide content of the particles as well.

The stability of nanoformulation was assessed by measuring the size and bosutinib content in PBS at 37 °C and over 48 h, which is the maximum incubation time used for the in vitro experiments in this study (Fig. 1C, D). Bosutinib-HDL NPs were stable both in terms of size and bosutinib content at the 24 h timepoint. No significant change in the size of bosutinib-HDL NPs was observed at the 48 h timepoint. However, the bosutinib content was reduced to 81.9 ± 17.3% of the initial content which shows some degree of bosutinib payload release. It should be noted that the stability and structural assembly of bosutinib-HDL NPs in vivo may be different from that in PBS as they can be affected by the presence of natural lipoproteins, potentially resulting in the remodelling of HDL NPs.

After the initial optimisation and characterisation of the bosutinib-HDL nanoformulation, its in vitro radiosensitisation properties were evaluated in UM-SCC-1 HNSCC cells. We have previously validated the expression of SR-B1 receptors in UM-SCC-1 and the 22A peptide-mediated uptake of HDL NPs [34]. HNSCC is often associated with dysfunctional *P53* pathways and is therefore critically dependent on the G_2_/M checkpoint. Chemoradiotherapy is frequently administered in patients with locally advanced HNSCC but relapse occurs within 2 years of treatment in approximately 50% of the cases [35–37]. The severe side effects associated with intense chemoradiation often lead to interruption or even discontinuation of treatment. In addition, the survival benefit is also limited to 4.5% at 5 years which emphasizes the critical need for the development of better radiosensitisation strategies [38].

**Fig. 2:**
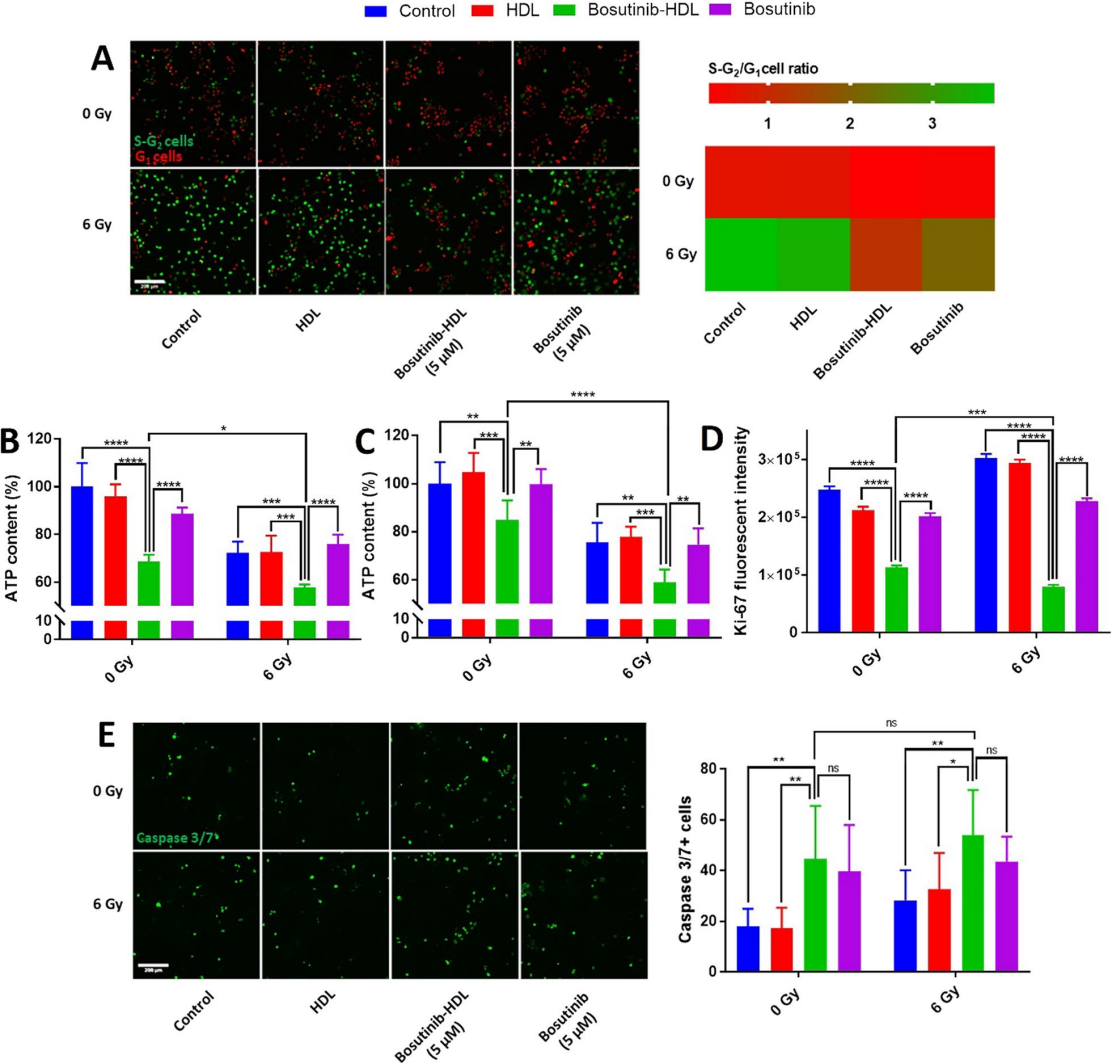
In vitro radiosensitisation properties of bosutinib-HDL NPs. **A** Cell cycle analysis using fucci-UM-SCC-1 cells. Cells were imaged 24 h after radiation treatment (n = 8). The scale bar is 200 µm. Data is represented as the mean in the graph. **B** ATP content in UM-SCC-1 cells measured by CellTiter-Glo^®^ assay 48 h after radiation (n = 5). **C** ATP content in U87 cells measured by CellTiter-Glo^®^ assay 72 h after radiation (n = 6). **D** Ki-67 expression in UM-SCC-1 cells 48 h after radiation. Data is represented as mean ± standard error of mean. **E** Effect of bosutinib-HDL NPs on apoptosis in UM-SCC-1 cells. Caspase 3/7 activity 24 h after radiation (n = 7, 8). The scale bar is 200 µm. Two way ANOVA and Tukey multiple comparisons test were used for statistical analysis (α = 0.05)

The effects of the bosutinib-HDL nanoformulation on the cell cycle pattern were first determined. Cell cycle checkpoints are key mediators to maintain the cellular genetic integrity after radiation treatments [39]. Previous reports show that bosutinib is a potent cell cycle checkpoint inhibitor which inhibits WEE kinase, a key mediator of the G_2_/M checkpoint [5]. The effect of bosutinib delivered by the HDL nanoformulation on cell cycle pattern following irradiation was analysed using fucci cell cycle indicator transfected UM-SCC-1 cells. Fucci reporter transfected cells emit red or green fluorescence in G_1_ and S-G_2_ phases, respectively. The majority of fucci-UM-SCC-1 cells were arrested at the G_2_/M cell cycle checkpoint 24 h after 6 Gy irradiation, as indicated by a relative increase in green fluorescent (S-G_2_) cells corresponding to a high S-G_2_ cells/G_1_ cells ratio (3.8 ± 1.2) in contrast to cells in the unirradiated control group (0.6 ± 0.1) (Fig. 2A). The radiation induced G_2_/M cell cycle arrest prevents cancer cells from progressing into mitosis while harbouring a significant burden of radiation induced DNA damages. Pre-treatment with the bosutinib-HDL NPs (5 µM) effectively disrupted the radiation induced G_2_/M cell cycle arrest, yielding a S-G_2_ cells/G_1_ cells ratio of 1.2 ± 0.2. The cell cycle disruption induced by the bosutinib-HDL NPs significantly outperformed an equivalent dose of free bosutinib (S-G_2_ cells/G_1_ cells ratio = 2.1 ± 0.6). On the other hand, HDL NPs did not alter the cell cycle pattern of irradiated UM-SCC-1 cells (S-G_2_ cells/G_1_ cells ratio = 3.5 ± 0.8).

The viability, proliferation index and apoptotic pathways were further analysed to achieve a better understanding about the radiosensitisation profile of the bosutinib-HDL nanoformulation. 48 h after irradiation (6 Gy), the ATP content of the UM-SCC-1 cells was reduced to 72.2 ± 4.7% of the content of cells in the unirradiated control group (Fig. 2-B). Pre-treatment with the bosutinib-HDL NPs (5 µM) significantly reduced the ATP content in irradiated cells (57.8 ± 1.2%, p < 0.001), while no difference vs the irradiated control was observed for cells treated with free bosutinib at this concentration (75.8 ± 4.0%). Cells treated with the bosutinib-HDL NPs without radiation also displayed reduced viability (68.6 ± 2.8%). The same ATP content response pattern was observed for the U87 cell line (Fig. 2C).

To further validate these observations, immuno-staining for the well-established proliferation marker ki-67 was performed (Fig. 2D). In line with the data yielded by the CellTiter-Glo^®^ assay, ki-67 expression was significantly reduced by pre-treatment of irradiated/nonirradiated UM-SCC-1 cells with the bosutinib-HDL nanoformulation. Again, the decrease in ki-67 expression was significantly more pronounced for cells treated with the combination bosutinib-HDL NPs and radiation (p < 0.001 vs non-irradiated and bosutinib-HDL NPs treated group; p < 0.0001 vs irradiated only group), confirming its impact on cell proliferation. Interestingly, an increase in ki-67 expression was also observed in cells treated with radiation only compared to the unirradiated control cells (p < 0.0001). This may be attributed to the radiation induced G_2_/M cell cycle arrest as cellular levels of ki-67 fluctuate during different phases of the cell cycle and peak in the G_2_/M phase [40, 41]. In agreement with our observation, Takahashi et al*.* previously reported increases in both ki-67 expression and G_2_/M cell population following 15 Gy irradiation [42].

The effect of bosutinib-HDL NPs treatment on apoptosis was next investigated by measuring caspase 3/7 activity. As shown in Fig. 2E, the frequency of caspase 3/7^+^ cells increased significantly upon treatment with both bosutinib-HDL NPs and free bosutinib, irrespective of radiation. Despite significant radiation induced cell cycle arrest at this point (24 h after radiation), no significant increase in the number of caspase 3/7 positive cells was observed in the radiation only control group. Interestingly, unlike cell cycle and viability analyses, the apoptotic response of bosutinib-HDL NPs and free bosutinib treated groups were comparable which warrants further investigations including measuring caspase 3/7 activity at later time points.

The data conclusively demonstrates potent radiosensitisation activity of the bosutinib-HDL nanoformulation in vitro, which could be attributed to disruption of the G_2_/M cell cycle checkpoint upon irradiation. The cell cycle disruption coincided with lower metabolic activity and an increased rate of apoptosis. It is noteworthy that the radiosensitisation activity of bosutinib is likely more complex as other mechanisms have been reported such as the downregulation of mediators involved in DNA damage response [6]. In addition, in cells treated with bosutinib-HDL NPs but not irradiated, reduced metabolic activity and increased apoptosis rate were observed despite a lack of significant cell cycle effect. This is in line with previously reported epidermal growth factor receptor (EGFR) inhibitory effects for bosutinib in head and neck cancer cell lines with high phosphorylated EGFR protein levels [43]. Further studies are warranted to assess the long term radiosensitisation effects of the bosutinib formulation which could be done using more complex in vitro biological models such as cancer spheroids and/or organoids.

Importantly, the bosutinib HDL nanoformulation demonstrated more potent radiosensitisation and anticancer effects compared to free bosutinib, which can potentially be attributed to different cellular uptake and subcellular localization. Interestingly, the bosutinib uptake analysis showed that the bosutinib intracellular level was lower for the bosutinib-HDL nanoformulation (1.4 ± 0.1 µg) compared to the free bosutinib (2.9 ± 0.8 µg) (Additional file 1: Fig S2). Despite lower intra-cellular uptake, bosutinib HDL nanoformulation resulted in significantly enhanced biological effects in the radio-sensitisation experiments. This suggests that while the total uptake of bosutinib is lower when formulated within reconstituted HDL NPs compared to free drugs, bosutinib is more efficiently delivered to its biological targets including WEE kinase localized in the cell nucleus [44]. While free bosutinib mostly remains partitioned in the cellular membrane due to its high hydrophobicity, bosutinib formulated in HDL NPs is potentially more efficiently delivered through HDL specific uptake mechanisms. HDL NPs mainly bind to SR-B1 receptors which are overexpressed on the surface of cancer cells. The SR-B1 mediated binding of the HDL NPs was previously shown to result in the formation of a non-aqueous channel that facilitates direct delivery of the drug payload to the cytoplasm without internalization of the nanoparticle itself [45]. Due to the likely different intracellular transit and fate of bosutinib molecules upon HDL based cellular uptake, it is possible that bosutinib is metabolized by different pathways compared to free drug. Further analyses are warranted to investigate this point, for example measuring formed bosutinib metabolites.

**Fig. 3:**
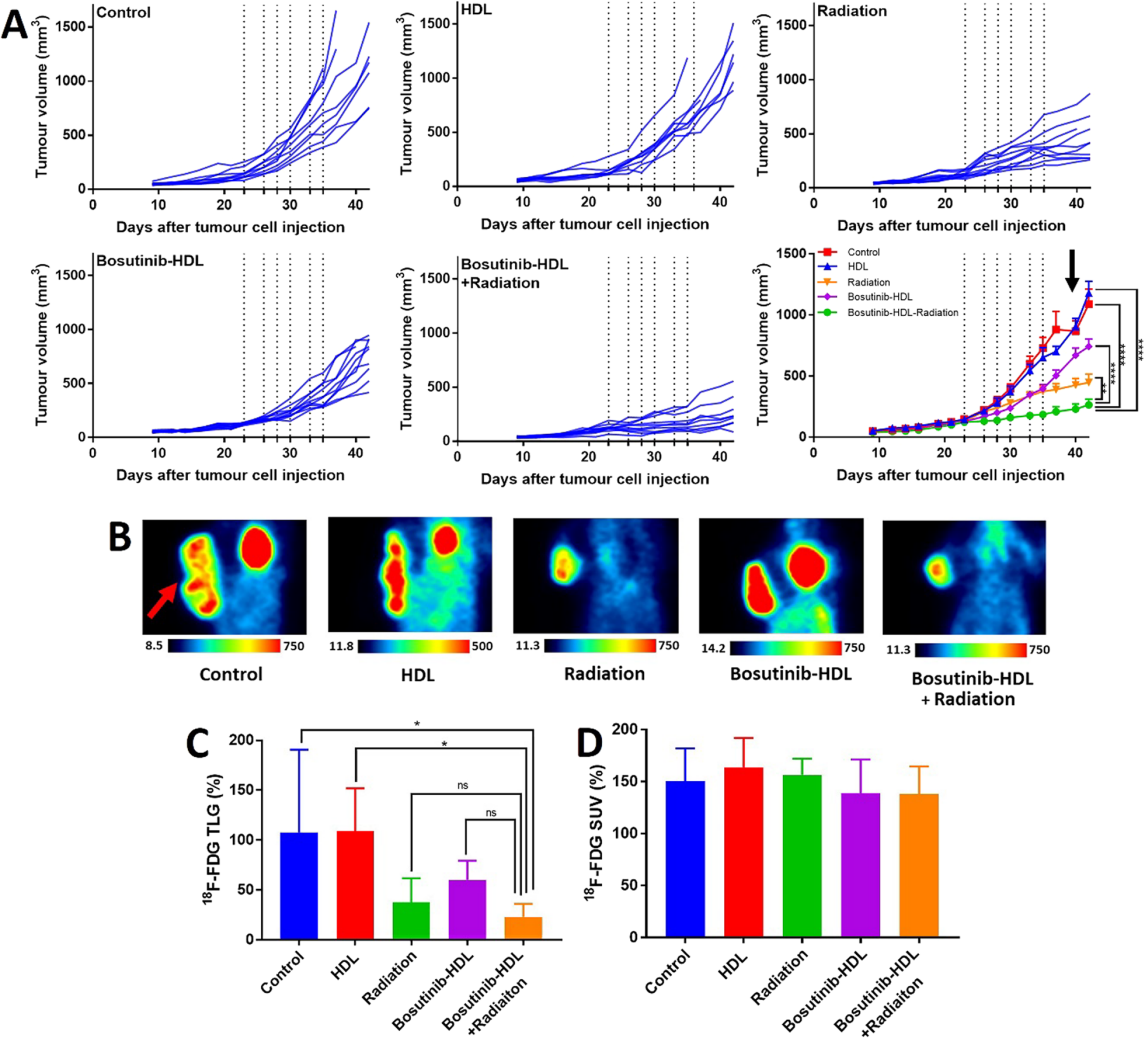
In vivo efficacy study of bosutinib-HDL NPs and radiation in heterotopic MOC-1 HNSCC tumour bearing murine model. **A** Tumour size during in vivo efficacy study. Bosutinib-HDL NPs and/or radiation treatment sessions are shown by dotted lines on the graph. Data is represented as mean ± standard error of the mean. Some mice were euthanised earlier than the study endpoint due to tumour ulceration complications, which is associated with fluctuations in the average tumour size in the control group (indicated with an arrow in the graph). Two way ANOVA and Tukey multiple comparisons test were used for statistical analysis (α = 0.05). **B** ^18^F-FDG PET images of mice approximately represent the average TLG% for each treatment group. The tumour location is depicted with an arrow (coronal section, tail and head are oriented toward the top and bottom of the image, respectively). ^18^F-FDG activity is represented as kBq/cc. **C**, **D** ^18^F-FDG TLG% and SUV% after 6 sessions of bosutinib-HDL NPs and radiation treatment (5 mice per treatment group). One way ANOVA and Tukey multiple comparisons test were used for statistical analysis (α = 0.05)

The radiosensitisation properties of bosutinib-HDL NPs were next evaluated in vivo in an immune-competent MOC-1 HNSCC tumour bearing murine model. The mice received 6 doses of the bosutinib-HDL nanoformulation (i.e. 6 × 20 mg/kg) and radiotherapy (i.e. 6 × 3 Gy). Tumour sizes were monitored as the main experimental endpoint of the study. Tumour growth inhibition was highest for the bosutinib-HDL NPs and radiation combination group, where the tumour growth was significantly slowed or stopped in some cases, yielding an average tumour size of 263 ± 142 mm^3^ at the study endpoint. The tumour size in the radiation only treated group that received cumulatively 18 Gy was 447 ± 210 mm^3^. Tumour sizes in both groups were significantly smaller than the control unirradiated group (1088 ± 302 mm^3^) (Fig. 3A). Treatment with HDL NPs had no significant effect on the tumour growth pattern. On the other hand, treatment with bosutinib-HDL NPs slowed down the tumour growth rate even in the absence of irradiation (741 ± 187 mm^3^). The observed efficacy of the combination of bosutinib-HDL NPs and radiation in controlling tumour growth rate is in line with disrupted cell cycle pattern and reduced proliferative index seen in the in vitro studies.

^18^F-FDG PET imaging was next performed after the last round of bosutinib-HDL NPs and radiation treatment to assess the tumour glucose uptake, which correlates to tumour size and metabolic activity. ^18^F-FDG TLG (%) measured in mice treated with either the bosutinib-HDL NPs only (no irradiation) or with radiation singly were 60.1 ± 19.1% and 37.6 ± 24.2%, respectively (Fig. 3B, C and Additional file 1: Fig S3). The lowest TLG% was observed for mice in the combination bosutinib-HDL NPs and radiation treated group (22.9 ± 13.2%), which was significantly lower compared to both control (107.7 ± 82.9%) and HDL (108.9 ± 43.0%) treated groups. In contrast to the volume based TLG%, the average glucose uptake rate (^18^F-FDG SUV %) was not significantly different among the various treatment groups (Fig. 3D). The observed pattern in ^18^F-FDG uptake is commonly seen in preclinical drug investigation [46, 47] and is potentially associated with features of the preclinical tumour model such as tumour vascular density, relative size of dead/necrotic zone and tumour ulceration which can all affect ^18^F-FDG tracer uptake. Heterotopic tumour bearing mice models are commonly used models for early stage in vivo preclinical screenings, specifically due to the extremely limited access to high end preclinical radiotherapy facilities that can conform the radiation dose to orthotopic head and neck tumours and spare sensitive organs in the cranial cavity. On the other hand, a notable limitation is that the excessively large tumour to body ratios (in some cases with tumours as large as 5–10% of mice body weight) in these models do not reliably mimic the clinical features of head and neck tumours.

**Fig. 4:**
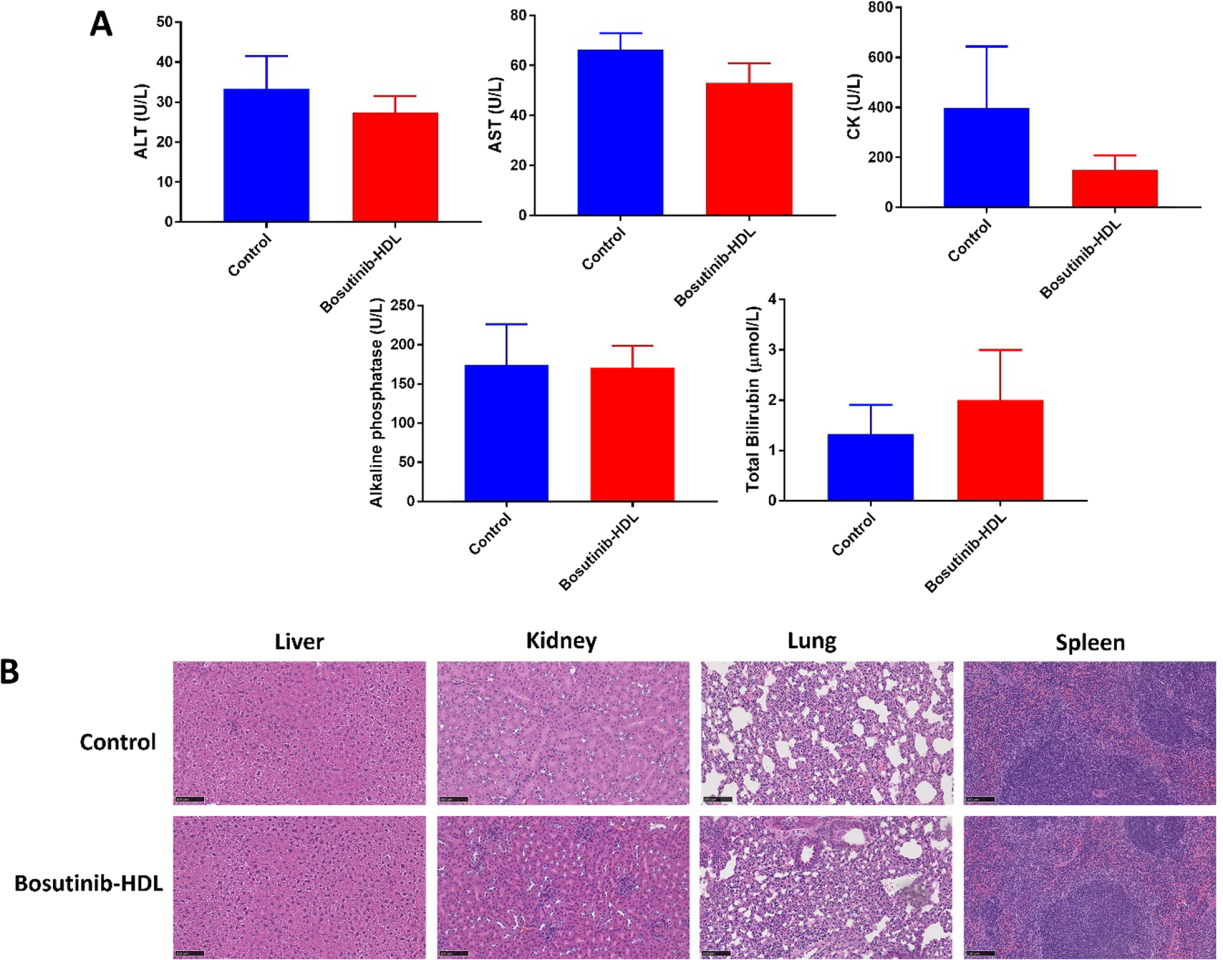
Toxicity study in healthy (non-tumour-bearing) mice. **A** Blood biochemical markers (n = 3) and **B** Hematoxylin and eosin staining of key organs, 2 weeks after receiving bosutinib-HDL NPs (20 mg/kg, IV)

In the absence of a gold-standard parenteral formulation for bosutinib, this study did not assess in vivo the therapeutic benefits of the HDL nanoformulation compared to the free drug. In most previous studies, bosutinib was administered orally and via very different dosing schedules compared to this study [48]. Bieerkehazhi et al. investigated the efficacy of intraperitoneal injection of bosutinib in a neuroblastoma preclinical model and reported a significant reduction in the tumour weight [49]. Mice were treated with a 30 mg/kg dose of bosutinib for 21 days which is a relatively higher dose compared to this study (6 doses of 20 mg/kg, IV). Such aggressive treatment raises concerns about off-target side-effects, which remains a major limitation in the clinical use of many orally administrated tyrosine kinase inhibitors such as bosutinib. To investigate this question, we next assessed the potential off-target effects associated to the bosutinib HDL nanoformulation. No significant adverse events were observed in treated mice during the efficacy study based on weight measurements (3 times a week, Additional file 1: Fig S4) and daily health monitoring. In addition, a more detailed toxicity study in healthy (non-tumour-bearing) mice showed no significant change in relevant blood biochemical markers, namely alanine aminotransferase (ALT), aspartate aminotransferase (AST), creatine kinase (CK), alkaline phosphatase (ALP) and total bilirubin, in the bosutinib-HDL NPs treated group versus the control group (Fig. 4A). To further assess potential off-target toxicity, the liver, kidney, lung and spleen were resected two weeks after treatment with the nanoformulation and stained with hematoxylin and eosin, before being examined for tissue inflammatory reactions. As shown in Fig. 4B, no noticeable pathological changes were observed in any of these organs for mice treated with the bosutinib formulation compared to untreated mice. All sections presented normal tissue morphologies, and no evidence of signs of necrosis or apoptosis was observed. While assessing the off-target toxicity of tyrosine kinase inhibitor in preclinical animal model is challenging, this data combined to the excellent tumour control afforded by the bosutinib HDL NPs validates the formulation design. Accumulation of HDL NPs in steroidogenic organs (e.g. reproductive organs) with high levels of SR-B1 expression can potentially be expected. Due to the significance of any potential side effects in these organs, more detailed toxicity studies combined with multiple dose treatment plans are required to fully validate the safety of the bosutinib HDL nanoformulation.

**Fig. 5:**
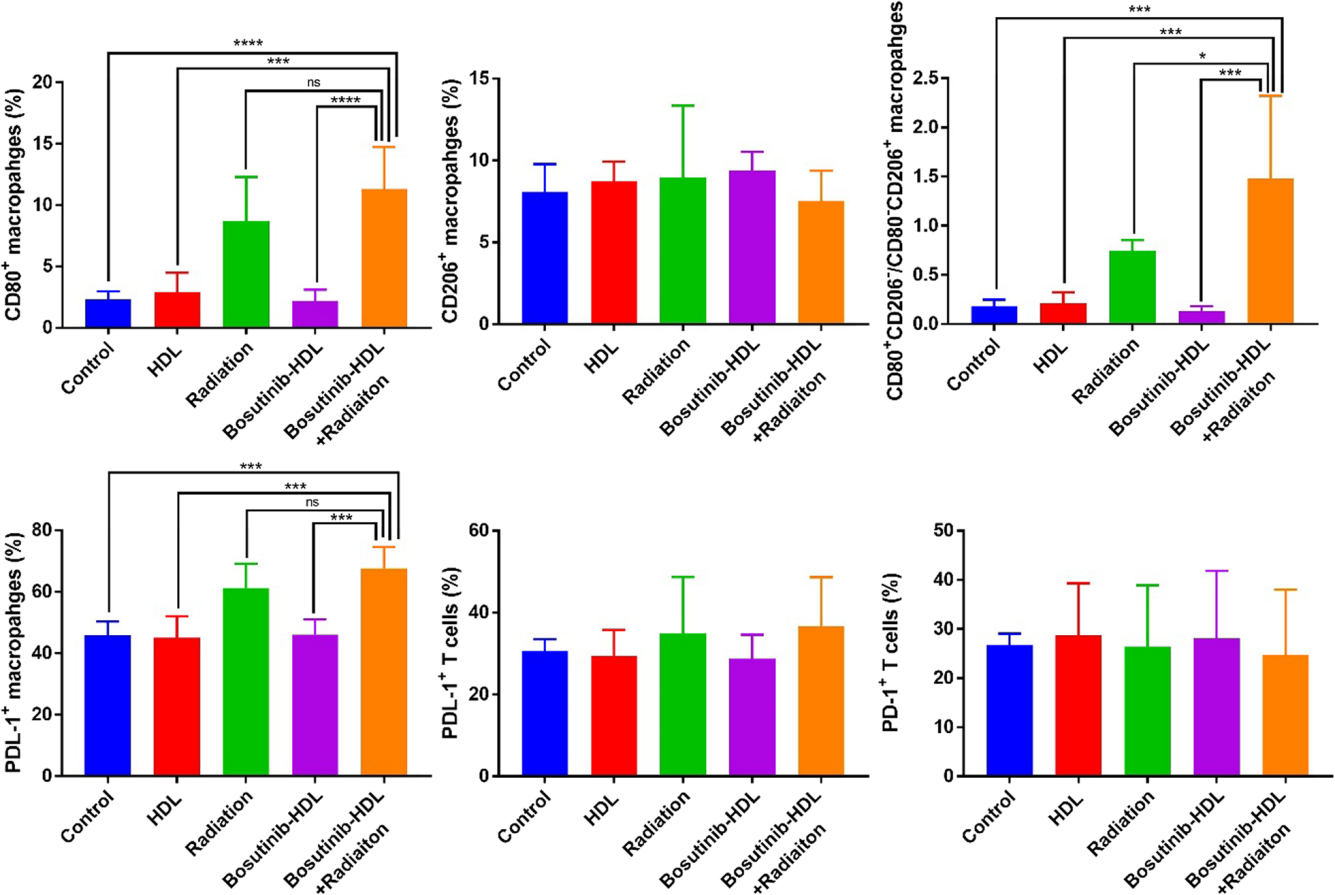
Flow cytometric analyses of immune cells in MOC-1 HNSCC tumours. Flow cytometric characterisation of TAMs phenotypes and exhaustion marker in TAMs and tumour infiltrating T cells at the study endpoint (5 mice per treatment group). One way ANOVA and Tukey multiple comparisons test were used for statistical analysis (α = 0.05)

Finally, towards ascertaining the mechanisms underpinning the anti-tumour effects of the bosutinib nanoformulation observed in vivo, we investigated its effects on the immune-landscape of resected tumours. The immune response after radiotherapy is a key contributing factor to radiotherapy efficacy or failure, due to the complex and somewhat opposite effects of radiation on the tumour immune landscape [50]. The immuno-competent nature of the C57BL/6 syngeneic model used in this study provided the opportunity to investigate the radiation induced immune response after bosutinib-HDL NPs treatment. At the experimental endpoint, tumour tissues were harvested and stained with an antibody cocktail to analyse the functional status of immune cells in the tumour microenvironment, namely TAMs and tumour infiltrating T cells. As expected, irradiation shifted the population of TAMs towards more proinflammatory phenotypes (CD80^+^CD206^−^/CD80^−^CD206^+^ = 0.7 ± 0.1) compared to the control group (CD80^+^CD206^−^/CD80^−^CD206^+^ = 0.2 ± 0.1) (Fig. 5). The polarisation of TAMs towards more proinflammatory phenotypes is potentially due to the enhanced exposure of tumour antigens after irradiation [51]. The combination of bosutinib-HDL NPs and radiation further increased the population of proinflammatory TAMs (CD80^+^CD206^−^/CD80^−^CD206^+^ = 1.5 ± 0.8), suggesting a substantial immuno-radiosensitisation function of the bosutinib nanoformulation. The enhanced levels of proinflammatory TAMs can be attributed to immunogenic cell death induced by the bosutinib-HDL NP and radiation combination, which further increases the presentation of tumour antigens. In line with this observation, AZD-1775, another WEE kinase inhibitor, was associated with accelerated maturation of antigen presenting dendritic cells [52]. In addition, AZD-1775 also decreased levels of CD163 anti-inflammatory marker in macrophages [53].

Despite the increased population of proinflammatory TAMs in the radiation only and bosutinib-HDL NPs/radiation combination treatment groups, an increase in expression of the PDL-1 exhaustion marker, was measured in TAMs and to a lesser extent in tumour infiltrating T cells. PDL-1 expression in TAMs increased significantly from 45.8 ± 4.5% in the control group compared to 61.2 ± 7.9%, and 67.6 ± 7.0 in the radiation only and radiation/bosutinib-HDL NPs combination treated groups, respectively. The increased expression of exhaustion markers is one of the main immunological complications after radiotherapy [54, 55], with the potential to decrease antitumour immunity. On the other hand, this data suggests that the therapeutic efficacy of the combination of bosutinib-HDL nanoformulation and radiotherapy could be further increased with the addition of an immune check point inhibitor targeting the PDL-1/PD-1 pathway.

## Conclusion

Kinase inhibitors targeting the cell cycle such as bosutinib have potent anticancer effects including radiosensitisation. However, the presence of significant off-target toxicities at doses required to reach effective intratumoural concentration is a major obstacle to implementation. To address this issue, we developed a bosutinib nanoformulation harnessing the excellent safety profile and inherent tumour homing properties of HDL NPs. Under optimal formulation conditions, very high bosutinib loading was achieved, which translated in potent radiosensitisation and more generally anticancer effect in vitro. These effects were ascribed to disruption of the G_2_/M cell cycle arrest upon irradiation. The bosutinib-HDL nanoformulation significantly outperformed equivalent doses of the free bosutinib. In vivo efficacy study in a syngeneic HNSCC murine model confirmed that pre-treatment with the bosutinib-HDL nanoformulation significantly increased the effect of radiation, as shown by substantially decreased tumour growth rate. In addition, the combination of bosutinib-HDL NPs and radiation shifted the polarization of TAMs towards a more proinflammatory phenotype, which potentially contributed to the observed effect. Altogether, the data presented here supports the potential of HDL nanoformulation of bosutinib and warrants further investigation aimed at ascertaining its therapeutic index and safety profile compared to current oral formulations of this potent tyrosine kinase inhibitor. The HDL nanoformulation also offers opportunities in the context of personalized medicine through the characterization of relevant receptors tumoural expression levels (e.g. SR-B1) in patients. From a translational point of view, recent successes in large-scale manufacturing of lipid nanoformulations such as COVID-19 vaccines provide a blueprint for scaled-up manufacturing of drug-loaded HDL nanoformulations.

## Supplementary Information

**Additional file 1**: **Figure S1**. Chemical structure of bosutinib. **Figure S2**. The intracellular level of bosutinib after 5 h incubation with either bosutinib-HDL NPs or free bosutinib (10 μM). Unpaired t test was used for statistical analysis (α=0.05). **Figure S3**. 18F-FDG PET images of mice. 18F-FDG PET imaging was performed after 6 sessions of bosutinib-HDL NPs and radiation treatment (5 mice per treatment group). The tumour location is depicted with an arrow (coronal section, tail and head are oriented toward top and bottom of image, respectively). 18F-FDG activity is represented as kBq/cc. **Figure S4**. Weight of mice during the *in vivo *study. The weight of mice was measured 3 times per week during period of the study. **Table S1**. Antibody cocktail for flow cytometric analyses.

## Data Availability

All data needed to evaluate the conclusions in the paper are present in the paper and/or the Additional files. Additional data related to this paper are available upon requests to the authors.
