# Supplementary material for: Bosutinib high density lipoprotein nanoformulation has potent tumour radiosensitisation effects
Source: J Nanobiotechnology. 2023 Mar 21;21:102. doi: 10.1186/s12951-023-01848-9 (PMC10028769; doi:10.1186/s12951-023-01848-9)
Supplement: Supplementary file 1 — Additional file 1: Figure S1. Chemical structure of bosutinib. Figure S2. The intracellular level of bosutinib after 5 h incubation with either bosutinib-HDL NPs or free bosutinib (10 μM). Unpaired t test was used for statistical analysis (α=0.05). Figure S3. 18F-FDG PET images of mice. 18F-FDG PET imaging was performed after 6 sessions of bosutinib-HDL NPs and radiation treatment (5 mice per treatment group). The tumour location is depicted with an arrow (coronal section, tail and head are oriented toward top and bottom of image, respectively). 18F-FDG activity is represented as kBq/cc. Figure S4. Weight of mice during the in vivo study. The weight of mice was measured 3 times per week during period of the study. Table S1. Antibody cocktail for flow cytometric analyses. [file 12951_2023_1848_MOESM1_ESM.pdf]

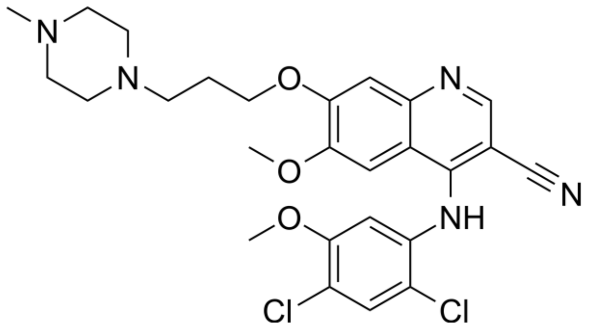

Figure S1. Chemical structure of bosutinib.

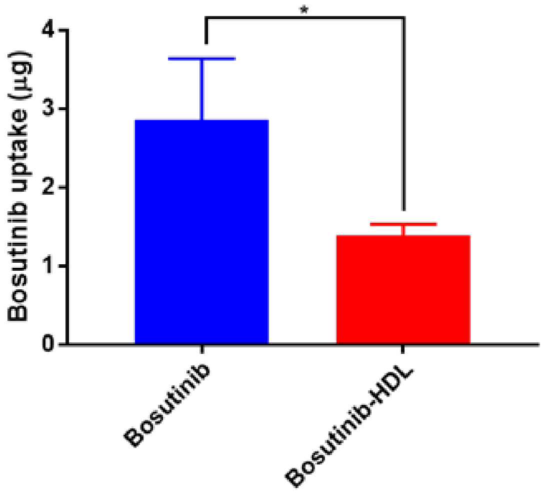

Figure S2. The intracellular level of bosutinib after 5 h incubation with either bosutinib-HDL NPs or free bosutinib (10 µM). Unpaired t test was used for statistical analysis (α=0.05).

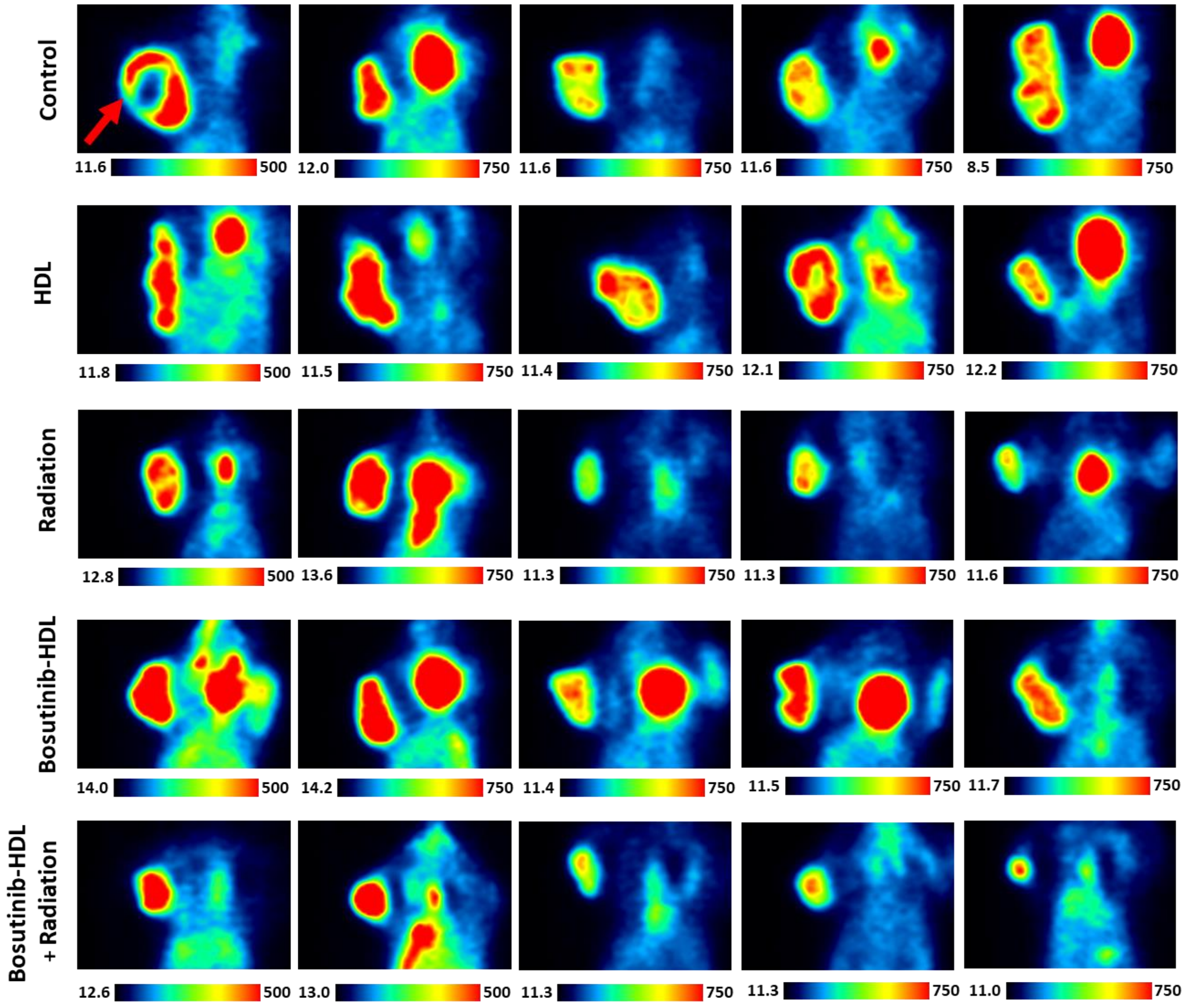

Figure S3: ^{18}F-FDG PET images of mice. ^{18}F-FDG PET imaging was performed after 6 sessions of bosutinib-HDL NPs and radiation treatment (5 mice per treatment group). The tumour location is depicted with an arrow (coronal section, tail and head are oriented toward top and bottom of image, respectively). ^{18}F-FDG activity is represented as kBq/cc.

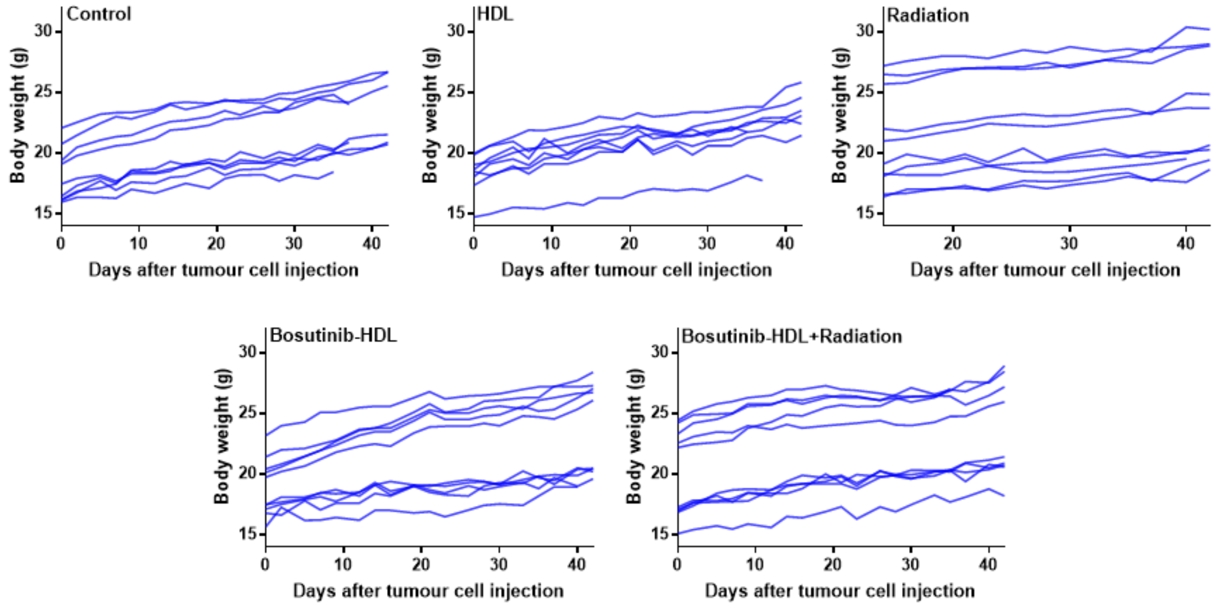

Figure S4. Weight of mice during the *in vivo* study. The weight of mice was measured 3 times per week during period of the study.

Table S1. Antibody cocktail for flow cytometric analyses.

Antibody	Clone	Fluorophore
Ms CD45	30-F11	BUV395
Ms CD80	16-10A1	BV421
Ms F4/80	T45-2342	BV711
Ms CD279 (PD-1)	RMP1-30	BB515
Ms CD274 (PDL-1)	MIH5	PE
Ms CD3e	145-2C11	PE-Cy7
Ms CD206	MR5D3	Alexa 647
